# Effects of a psychosocial intervention at one-year follow-up in a PREDIMED-plus sample with obesity and metabolic syndrome

**DOI:** 10.1038/s41598-021-88298-1

**Published:** 2021-04-28

**Authors:** Núria Mallorquí-Bagué, María Lozano-Madrid, Cristina Vintró-Alcaraz, Laura Forcano, Andrés Díaz-López, Ana Galera, Rebeca Fernández-Carrión, Roser Granero, Susana Jiménez-Murcia, Dolores Corella, Xavier Pintó, Aida Cuenca-Royo, Mònica Bulló, Jordi Salas-Salvadó, Rafael de la Torre, Fernando Fernández-Aranda

**Affiliations:** 1 Addictive Behaviours Unit, Department of Psychiatry, Hospital de la Santa Creu i Sant Pau, Biomedical Research Institute Sant Pau (IIB Sant Pau), Barcelona, Spain; 2 Department of Psychiatry, University Hospital of Bellvitge-IDIBELL, Feixa Llarga S/N, L’Hospitalet del Llobregat, 08907 Barcelona, Spain; 3 Consorcio CIBER, M.P. Fisiopatología de la Obesidad y Nutrición (CIBERObn), Instituto de Salud Carlos III (ISCIII), Madrid, Spain; 4 Integrative Pharmacology and Neurosciences Systems, Institut Hospital del Mar d’Investigacions Mèdiques (IMIM), Dr. Aiguder 88, 08003 Barcelona, Spain; 5 Universitat Rovira i Virgili, Departament de Bioquímica i Biotecnologia, Unitat de Nutrició Humana, Reus, Spain; 6 Lipids and Vascular Risk Unit, Internal Medicine, University Hospital of Bellvitge, Hospitalet de Llobregat, Barcelona, Spain; 7 Department of Preventive Medicine, University of Valencia, Valencia, Spain; 8 Departament de Psicobiologia i Metodologia, Universitat Autònoma de Barcelona, Cerdanyola del Vallès, Barcelona, Spain; 9 Department of Clinical Sciences, School of Medicine and Health Sciences, University of Barcelona, Hospitalet de Llobregat, Barcelona, Spain; 10 Institut d’Investigació Sanitària Pere Virgili (IISPV), Reus, Spain; 11 Nutrition Unit, University Hospital of Sant Joan de Reus, Reus, Spain; 12 Departament de Ciències, Experimentals i de la Salut Universitat Pompeu Fabra (CEXS-UPF), Barcelona, Spain; 13 Serra Hunter Fellow, Universitat Rovira i Virgili (URV), Reus, Spain

**Keywords:** Psychology, Human behaviour

## Abstract

This study examines if overweight/obesity are related to higher impulsivity, food addiction and depressive symptoms, and if these variables could be modified after 1 year of a multimodal intervention (diet, physical activity, psychosocial support). 342 adults (55–75 years) with overweight/obesity and metabolic syndrome (MetS) from the PREDIMED-Plus Cognition study were randomized to the intervention or to the control group (lifestyle recommendations). Cognitive and psychopathological assessments were performed at baseline and after 1-year follow-up. At baseline, higher impulsivity was linked to higher food addiction and depressive symptoms, but not to body mass index (BMI). Food addiction not only predicted higher BMI and depressive symptoms, but also achieved a mediational role between impulsivity and BMI/depressive symptoms. After 1 year, patients in both groups reported significant decreases in BMI, food addiction and impulsivity. BMI reduction and impulsivity improvements were higher in the intervention group. Higher BMI decrease was achieved in individuals with lower impulsivity. Higher scores in food addiction were also related to greater post-treatment impulsivity. To conclude, overweight/obesity are related to higher impulsivity, food addiction and depressive symptoms in mid/old age individuals with MetS. Our results also highlight the modifiable nature of the studied variables and the interest of promoting multimodal interventions within this population.

## Introduction

Overweight and obesity are among the greatest health challenges of this century. They significantly increase the risk of death and chronic diseases such as metabolic syndrome (MetS), which is a cluster of cardiovascular risk factors that includes abdominal obesity, insulin resistance/diabetes, hypertension, and atherogenic dyslipidemia^1^. This subject is of big concern for the population aged 60 or over, as in this age range the prevalence of obesity is as high as 84%^2^. Overweight/obesity have been associated with different psychological factors, such as impulsivity^3^, food addiction^4,5^ and psychological wellbeing^6^.

Impulsivity is a pattern of undercontrolled behaviour or a tendency to act out in response to impulses^7^ and it can be conceptualized along three different domains^8–10^: impulsive personality traits, deficits in inhibitory control and impulsive choice or decision-making. Higher body mass index (BMI) and obesity have been linked to poorer inhibitory control^11,12^, impaired decision-making^13–17^ and certain impulsive traits^5,18^. In the older age population, there is still little evidence on the association between impulsivity and body weight. The few current studies in inhibitory control have shown unconclusive results, with some pointing to reduced inhibitory control in older adults with higher BMI^19–21^, but others indicating normal parameters in this population^22^. Regarding impulsive choice, there is even less evidence^23^, with only a recent study showing that increased reward sensitivity may characterize higher BMI throughout aging^22^.

Food addiction (FA) is a developing construct, which refers to an overconsumption of highly palatable food sustained by addictive processes^24^. It is highly prevalent (25–30%) in individuals with overweight/obesity^25^ and it is associated with high levels of impulsivity^3,26–28^. Besides, FA has been described as a mediator of the association between impulsivity and obesity^5^ and postulated to share similar neural mechanisms with other addictions (e.g.: greater activation of striatal regions to food/drug cues)^29–31^. Nevertheless, there is still a lack of studies examining these associations in elderly population with MetS.

Psychological health appears to worsen with the increasing severity of obesity^32,33^ and individuals with overweight/obesity carry a high prevalence of different psychological comorbidities (e.g.: mood and anxiety disorders)^34–37^. Some studies highlight the link between obesity and depression in adulthood, placing depression as a risk factor for developing obesity^38,39^ especially among females^40^. Besides, this population often suffers from prejudice and feelings of dislike about their bodies and appearance, which commonly lead to greater levels of depressive symptoms^41^. For this reason, some researchers uphold that the link between these two factors is bidirectional^38^ yet this is not conclusive and some contrary findings have also been reported^42^.

Finally, different studies have explored how changes in lifestyle, such as increased physical activity and healthier eating habits, may reduce body weight and the risk of cardiovascular diseases, type 2 diabetes and MetS^43–50^. In fact, these positive effects can be observed not only on BMI but also on impulsivity, FA and psychological health^51–58^. Nevertheless, longitudinal studies that consider all previously mentioned variables while controlling for relevant confounders are needed to increase the validity of current knowledge.

The first aim of this study was to examine if overweight and obesity in mid-old age individuals with MetS are related to higher impulsivity, food addiction and depressive symptoms. We hypothesized that impulsivity features would predict higher BMI, FA and depressive symptoms. The second aim was to longitudinally explore through a randomized clinical trial if these variables could be modified after 1 year of a multimodal psychosocial intervention (based on an energy-restricted diet, physical activity promotion and behavioural-motivational support). By doing so, we aim to not only better describe the psychological factors related to overweight/obesity but also to test the effectivity of the treatment when dealing with them. We hypothesize that the multimodal intervention, compared to the control intervention, would higher reduce BMI, FA, depressive symptoms as well as impulsivity. Our last hypothesis is that the severity of impulsivity and FA at baseline would predict the extent of BMI pre-post change.

## Methods

### Design

This is a cross-sectional and longitudinal data analysis from baseline and 1-year follow-up within the frame of the PREDIMED-Plus study (Cognition Subprogram), a multicentre, randomized, parallel-group, primary prevention clinical trial conducted in Spain to assess the effect of an intensive weight loss intervention program based on an energy-restricted traditional Mediterranean diet, physical activity promotion and behavioural support^59,60^. The design and methods have been published^59,60^ and the study protocol is available at http://www.predimedplus.com/. This study was registered at the International Standard Randomized Controlled Trial (ISRCT; http://www.isrctn.com/ISRCTN89898870, Date applied 28/05/2014, Date assigned 24/07/2014). Cognitive function, quality of life, and psychological and neuropsychological scores are secondary outcomes of the study.

### Participants

The study was comprised of 489 participants who were randomly assigned to control group (n = 247) or intervention group (n = 242). 147 participants were excluded from analysis given that their assessments had no complete data in relation to the outcome variables studied. Final analyses were conducted with 342 participants (167 females). Control and intervention group consisted of 179 and 163 participants respectively (see the Supplementary Figure S1 for the Consort flow diagram). Participants were recruited September 2014 to October 2016 from Primary Health Care facilities belonging to the National Health System associated to four centres from different universities, teaching hospitals and research institutes in Spain participating in the PREDIMED-Plus cognition subprogram: Universitat Rovira i Virgili (Tarragona), Universidad de Valencia (Valencia), Institut Hospital del Mar d’Investigacions Mèdiques-IMIM (Barcelona), and Bellvitge University Hospital-IDIBELL (Barcelona). The eligible participants were community-dwelling adults (aged between 55 and 75 years in case of men; and between 60 and 75 years in women), free of cardiovascular disease (CVD) at enrolment, with a BMI of 27–40 kg/m^2^, and harbouring the MetS^61^. Further details of the inclusion and exclusion criteria can be found elsewhere^59,60^. Participants included in the current analysis were part of the PREDIMED-Plus and were explored with an extended cognitive protocol. Data collected assessed cognitive domains related with impulsivity and food addiction. Depressive symptoms, as well as detailed specification of BMI, physical conditions and sociodemographic data were collected in the frame of the PREDIMED-Plus study. All participants provided written informed consent, and the study protocol and procedures were approved according to the ethical standards of the Declaration of Helsinki by the Research Ethics Committees from all the participating institutions: CEIC Hospital Universitari Sant Joan de Reus (13-7-25/7proj2), CEIm-PSMAR (2019/8612/I), CEIC Hospital Universitari de Bellvitge (PR240/13), Institutional Review Board of Valencia University (H1373255532771).

### Measures

All participants were assessed at baseline and reassessed at 1-year follow-up. During these two assessments, the participants completed several self-reported questionnaires and a set of neuropsychological tests for evaluating impulsivity domains and decision making (namely Stroop Colour and Word Test—SCWT, Conner’s Continuous Performance Test—CPT and Iowa Gambling Task—IGT) and psychopathological factors (namely depression symptomatology and food addiction). At each visit, sociodemographic-clinical information, including age, weight, height, and other relevant indexes were also collected by means of a self-reported questionnaire and a hetero-administered clinical interview assessing medical conditions.

#### Clinical and psychometrical assessment:

The Beck Depression Inventory–II^62^ is a 21-item self-report measure for assessing the severity of depressive symptoms in adults and adolescents (ages from 13 to 80 years). The BDI-II reflects the diagnostic criteria for Major Depressive Disorder listed in the DSM-5^63^. Scores for each item range from 0 to 3; the total score is the sum of all responses. The Cronbach’s alpha in our sample shows a good internal consistency (α = 0.874).

The Yale Food Addiction Scale version 2.0^64,65^ is a 35 item self-report questionnaire scored on an eight-level Likert scale (from 0 = never to 7 = every day) to measure addictive food behaviours based on DSM-5 substance-related and addictive disorders criteria^63^. The YFAS 2.0 has been validated in a Spanish-speaking population, presenting excellent accuracy in discriminating between healthy control and eating-disorder subsamples (α = 0.75) and excellent internal reliability coefficient (α = 0.94). Two scores can be derived from the YAFS: (a) a continuous symptom count score that reflects the number of fulfilled diagnostic criteria (ranging from 0 to 11) and (b) a diagnosis of food addiction based on the number of symptoms and clinically significant impairment or distress. The Cronbach’s α value for the present study was 0.883.

The UPPS-P Impulsivity Scale^66^ is a 59-item questionnaire to assess five different features of impulsive behaviour: negative urgency, lack of perseverance, lack of premeditation, sensation seeking and positive urgency. The UPPS-P has satisfactory psychometric properties in terms of both convergent and discriminatory validity^67,68^. The Spanish adaptation of the scale has adequate psychometric properties^69^. The α values for the different UPPS-P scales in our sample are as follows: lack of premeditation (0.818), lack of perseverance (0.759), sensation seeking (0.846), positive urgency (0.897) and negative urgency (0.812).

#### Neuropsychological assessment

The Stroop Colour and Word Test^70,71^ is a widely employed task which assesses inhibitory control, that is, the cognitive ability to control dominant behavioural responses to stimuli. It involves three different lists: a word list with names of colours printed in black ink, a colour list including letter Xs printed in colour, and a colour-word list composed of names of colours in a colour ink that does not match the written name. Three final scores are obtained calculating the number of items read in 45 s. An interference score is also computed based on the three aforementioned lists, this score enables the assessment of individual’s cognitive flexibility and the ability to inhibit cognitive interference. Higher scores in this index mean better inhibitory control.

The Conner’s Continuous Performance Test, second edition^72^ is a computerized task in which participants have to press the space bar in response to visual stimuli (i.e., letters on the computer screen). This task has a duration of 14 min. The CPT-II provides information about the participants' errors (i.e. omissions and commissions), reaction time and response variability. All these measures are used to assess sustained attention and inhibitory control. Higher scores on the CPT-II indicate worse performance. High levels of impulsivity involve a fast Hit Reaction time (HRT) together with a high rate of Commissions^72^.

The Iowa Gambling Task^73,74^ is a computer-based task which enables to assess decision-making, which is proposed to be a measure of choice of impulsivity^75^. It comprises a total of 100 trials in which the participant has to select among four decks of cards (A, B, C, and D), a specified amount of play money is awarded afterward. The interspersed rewards amongst these decks are probabilistic punishments (monetary losses). The final aim of the task is to win as much money as possible trying to lose as less money as possible. In addition, they might choose cards from any deck, and switch decks at any time. The final score is obtained by subtracting the number of cards selected from decks A and B from the number of cards selected from decks C and D. These two last decks are advantageous as the punishments are smaller, while decks A and B are disadvantageous since the final loss is higher than the final gain. Higher scores implicate better performance on the task.

#### Anthropometric and biochemical measurements

Calibrated weighting scales were used to measure weight, while a wall-mounted stadiometer provided information about participants’ height. BMI was calculated though the following equation: BMI = kilograms/meters^2^.

### Clinical interventions

Participants randomized to the control group received usual lifestyle recommendations for the management of the MetS. They were recommended to follow a Mediterranean diet based on materials and results developed in the framework of the PREDIMED study^59,60^. The participants of the control group were offered sessions every 6 months, which consisted of an individual visit, a telephone call and a group session, all of them led by the team of dieticians of the PREDIMED-Plus study. No specific motivational advice for increasing physical activity or losing weight was provided.

Participants randomized to the intervention group (intensive lifestyle with a psychosocial approach) received an energy restricted traditional Mediterranean diet. Dietary intervention was associated with physical activity promotion and psychosocial (behavioural-motivational) support, with specific goals of weight loss, including self-monitoring and frequent monitoring throughout the study. These subjects participated in individual interviews (15–30 min) and motivational group sessions (30–45 min; maximum 20 participants) three times per month during the first year of the intervention. The level of compliance with the intervention was periodically monitored in order to adapt it if deemed necessary.Participants in both groups were provided with an allotment of extra-virgin olive oil (1 L/mo) and raw nuts (125 g/mo) for free. However, we recommended that all participants consume a total of 500 g/mo of mixed nuts.

Further information on the intervention procedures and goals were previously described elsewhere^59,76^.

### Statistical analysis

Statistical analysis was carried out with SPSS24 and Stata16 for Windows using the available database January 15^th^, 2019 PREDIMED-Plus Cognition subprogram database. Comparisons on the descriptive variables of the two intervention groups were implemented via chi-square (categorical variables) and *t* test tests (quantitative variables). Multiple regression analysis was used to test if the impulsivity measures predicted FA (YFAS-total score), BMI (kg/m^2^) and depressive symptoms (BDI total score) at baseline. These models were obtained in two blocks (each block representing one step): a) the first block entered and fixed the covariates sex, age, and education; and b) the second block used a stepwise procedure to automatically select the impulsivity measures which were significantly predicting each outcome. The incremental predictive capacity of impulsivity was estimated with the R^2^ change/increase.

Pre-post changes were tested with a mixed analysis of variance (ANOVA), defining time (pre-post) as the intra-subjects’ factor and intervention group (control-intervention) as the between-subjects factor. These analyses were adjusted by sex, age and education.

Multiple regression analysis was used to test the incremental predictive capacity of FA and impulsivity at baseline as well as group of treatment (control-intervention) on pre-post changes in BMI (kg/m^2^). These models were obtained in four blocks (each block representing one step): a) the first block entered and fixed the covariates sex, age, education, and BMI at baseline; b) the second block entered and fixed the covariate FA; and c) the third block used a stepwise procedure for automatically selecting the best predictors (from the set of impulsivity measures—UPPS-P, STROOP word/colours and interference, CPT commissions-omissions—hit reaction time) of the outcome; and d) the fourth block entered and fixed the treatment group. Incremental predictive capacity of each block was estimated with the increase/change in the R^2^ coefficient.

Path analysis (a special case of structural equation modelling: SEM) was implemented to analyse the underlying mechanisms explaining the mediational links (direct and indirect effects) among the variables of the study^77^. In this study, the maximum-likelihood estimation (MLE) method of parameter estimation was used and a latent variable was defined for the impulsivity trait considering the scores in the UPPS-P scales. Goodness-of-fit was considered for^78^: measuring root mean square error of approximation RMSEA < 0.08, Bentler’s Comparative Fit Index CFI > 0.90, Tucker-Lewis Index TLI > 0.90, and standardized root mean square residual SRMR < 0.10.

In this study, the increase of Type-I error due to multiple comparisons was controlled with the Finner-method (a familywise error rate stepwise procedure which has demonstrated higher power than the classical Bonferroni-method^79^).

## Results

### Characteristics of the participants

No statistical differences were found in sociodemographic data when compared participants included and those excluded due to missing values in the outcome variables (age: *F* = 0.52, *df* = 1/487, *p* = 0.472; sex: χ^2^ = 1.29, *df* = 1, *p* = 0.257; origin European vs other: χ^2^ = 1.19, *df* = 1, *p* = 0.275; civil status: χ^2^ = 0.34, *df* = 1, *p* = 0.559; education level: χ^2^ = 7.55, *df* = 4, *p* = 0.109; and employment status: χ^2^ = 0.72, *df* = 1, *p* = 0.395), BMI at baseline (*F* = 0.02, *df* = 1/487, *p* = 0.880) and psychometrical measures at baseline (UPPS-premeditation: *F* = 1.14, *df* = 1/487, *p* = 0.287; UPPS-perseverance: *F* = 0.55, *df* = 1/487, *p* = 0.458; UPPS-sensation seeking: *F* = 1.66, *df* = 1/487, *p* = 0.199; UPPS-positive urgency: *F* = 0.91, *df* = 1/487, *p* = 0.341; UPPS-negative urgency; *F* = 2.50, *df* = 1/487, *p* = 0.115; YFAS-total: *F* = 2.53, *df* = 1/487, *p* = 0.112; BDI-total: *F* = 1.31, *df* = 1/487, *p* = 0.254).

Table 1 includes the distribution of the sociodemographic features and the BMI at the baseline for the participants in this study. No baseline significant differences were observed between the two groups on age, education or BMI. Similarly, no statistical differences were found for the final sample of the two treatment arms on the sociodemographic or main general variables of the study (see Figure S1 for excluded participants due to missing data) (sex: χ^2^ = 1.29, *p* = 0.257; origin: χ^2^ = 1.19, *p* = 0.275; civil status: χ^2^ = 0.34, *p* = 0.559; school level: χ^2^ = 7.55, *p* = 0.109; employment status: χ^2^ = 0.72, *p* = 0.395; age: F = 0.52, *p* = 0.472; and BMI: F = 0.02, *p* = 0.880).

**Table 1:** Descriptive for the sample at baseline.

	Total (*n* = 342)		Control (*n* = 179)		Intervention (*n* = 163)		*χ*^*2*^	*df*	*p*
	*n*	%							
**Sex**									
Male	175	51.2	90	50.3	85	52.1	0.12	1	.730
Female	167	48.8	89	49.7	78	47.9			
**Civil status**									
Single	8	2.3	5	2.8	3	1.8	2.21	3	.702
Married	271	79.2	146	81.6	125	76.7			
Divorced-separated	28	8.2	12	6.7	16	9.8			
Widowed	35	10.2	16	8.9	19	11.7			
**School education**									
University (high)	40	11.7	26	14.5	14	8.6	5.68	4	.497
University (grade)	28	8.2	18	10.1	10	6.1			
Secondary	102	29.8	53	29.6	49	30.1			
Primary or less	172	50.3	82	45.8	90	55.2			
**Employment**									
Employed	67	19.6	34	19.0	33	20.2	3.61	4	.702
Work at home	27	7.9	12	6.7	15	9.2			
Retired	223	65.2	118	65.9	105	64.4			
Unemployed (incomes)	19	5.6	13	7.3	6	3.7			
Unemployed (no-incomes)	6	1.8	2	1.1	4	2.5			
**Group of weight**									
Over-weight	85	24.9	42	23.5	43	26.4	2.76	3	.702
Obesity I (BMI 30–35)	181	52.9	101	56.4	80	49.1			
Obesity II (BMI 35–40)	76	22.2	36	20.1	40	24.5			

	*Mean*	*SD*	*Mean*	*SD*	*Mean*	*SD*	*F*	*df*	*p*
Age (years-old)	65.24	4.65	65.12	4.64	65.37	4.68	0.25	1/340	.855
BMI (kg/m^2^)	32.55	3.34	32.52	3.20	32.58	3.50	0.03	1/340	.872

*SD* standard deviation. *Df* degrees of freedom. *BMI* body mass index.

#### Cross-sectional analysis

Table 2 contains the three multiple regressions estimating the predictive capacity of the impulsivity measures on FA scores (YFAS total), BMI and depressive symptoms (BDI total) at baseline. After adjusting by the participants’ sex, age and education, higher FA and depressive symptoms were observed on individuals with higher scores in the UPPS-P lack of perseverance and negative urgency impulsivity components. No significant predictors of BMI were found for the set of impulsivity measures. For these analyses, participants from both intervention and control groups were pooled.

**Table 2:** Predictive models of FA, BMI and depressive symptoms at baseline: multiple linear regressions (final models).

		Coefficients						Change statistics	
		B	SE	Beta	*p*	95%CI B		∆R^2^	*p*
**FA (YFAS total)**									
1^st^ block	Sex (0 = men; 1 = women)	0.27	0.13	.111	.045*	0.01	0.53	.022	.053
	Age (years-old)	− 0.01	0.01	− .037	.501	− 0.04	0.02		
	Education (years)	− 0.01	0.01	− .024	.667	− 0.03	0.02		
2^nd^ block	UPPS-P Lack perseverance	0.04	0.01	.151	.006*	0.01	0.07	.078	.001*
	UPPS-P Negative urgency	0.04	0.01	.199	< .001*	0.02	0.06		
**BMI (kg/m**^**2**^**)**									
1^st^ block	Sex (0 = men; 1 = women)	0.21	0.38	.031	.587	− 0.54	0.96	.002	.907
	Age (years-old)	0.01	0.04	.016	.776	− 0.07	0.09		
	Education (years)	0.02	0.04	.032	.584	− 0.06	0.10		
2^nd^ block (STEPWISE)	*No significant predictors*								
**Depressive symptoms (BDI total)**									
1^st^ block	Sex (0 = men; 1 = women)	3.84	0.72	.272	< .001*	2.42	5.25	.117	< .001*
	Age (years-old)	− 0.01	0.08	− .009	.852	− 0.17	0.14		
	Education (years)	− 0.11	0.07	− .078	.130	− 0.26	0.03		
2^nd^ block	UPPS-P Lack perseverance	0.26	0.07	.175	.001*	0.11	0.40	.112	< .001*
	UPPS-P Negative urgency	0.26	0.05	.244	< .001*	0.15	0.36		

*FA* food addiction, *BMI* body mass index.

*Bold: significant parameter (.05 level). ∆R^2^: R^2^ change. (*N* = 342).

**Figure 1:**
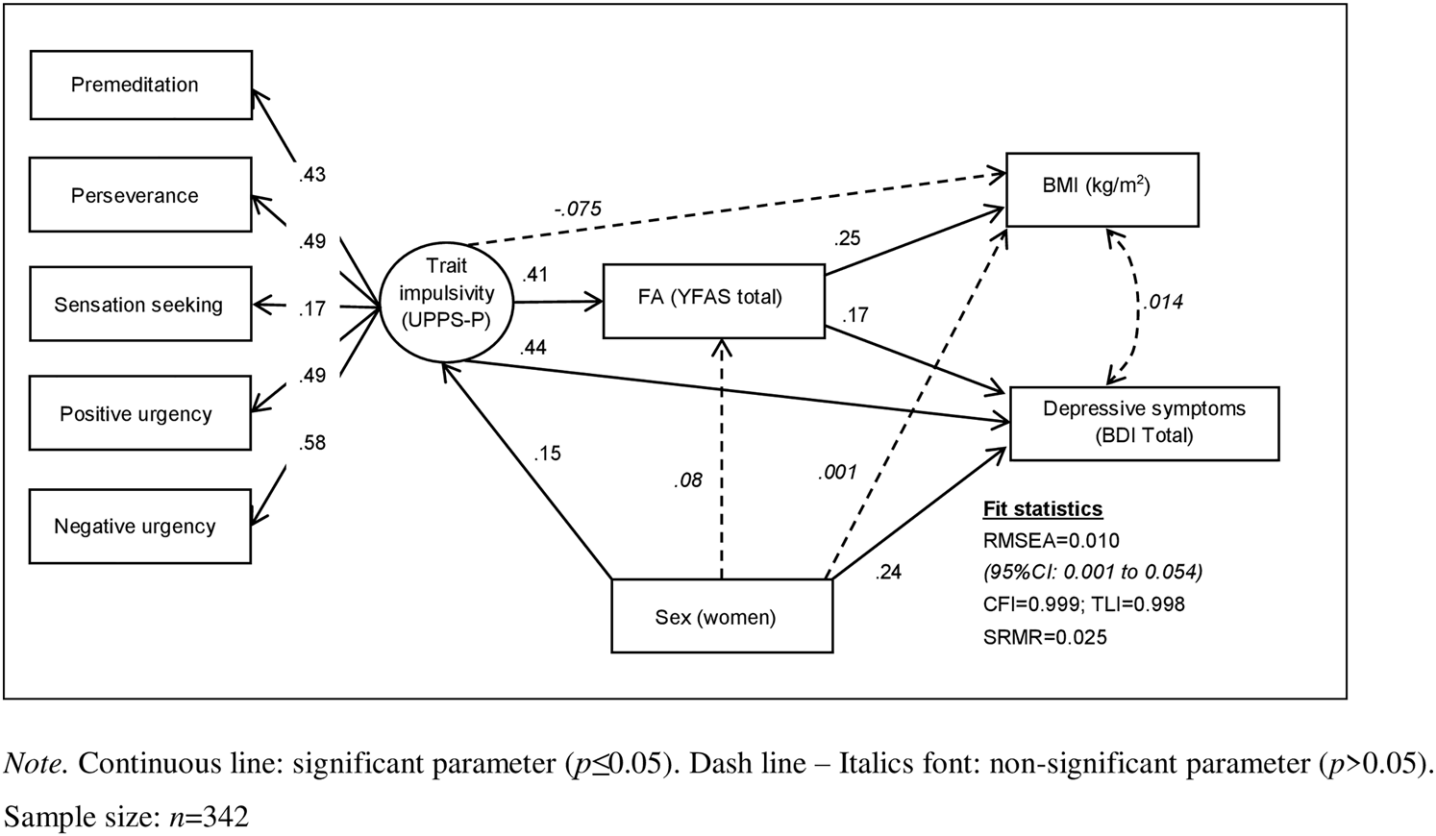
Path-diagram with the standardized coefficients in the SEM at baseline.

Figure 1 includes the path-diagram with the standardized coefficients obtained in the SEM with the measures at baseline (adequate fitting was achieved). The latent variable measuring trait impulsivity was significantly and positively defined by the set of the UPPS-P (the highest coefficient-weight was achieved for the negative urgency scale and the lowest for the sensation seeking). FA severity was positively associated with impulsivity. Higher BMI was only predicted by higher FA scores, whereas higher depressive symptoms were predicted by both higher FA scores and higher impulsivity. FA achieved a mediational role between the impulsivity levels and the BMI and BDI scores. Women significantly displayed increased impulsivity and the BDI scores, but there were no significant predictors of the YFAS or the BMI.

#### Longitudinal analysis after 1 year of intervention

Table 3 contains pre-post changes in each treatment group for the variables of the study (i.e.: impulsivity measures, FA and BMI) adjusted by sex, age and education. Patients from both groups presented a significant decrease in BMI and FA scores; although the decrease in BMI was significantly higher for the patients in the intervention group.

**Table 3:** Pre-post changes in the variables of the study: mixed ANOVA adjusted by sex, age and years of school education.

	Control (*n* = 179)				Intervention (*n* = 163)				Interaction		Factor: group (control vs exp)				Factor: time (pre vs post)			
	Pre		Post		Pre		Post		Group-by-time		Pre		Post		Control		Experim	
	Mean	SD	Mean	SD	Mean	SD	Mean	SD	F _(df=1/337)_	*p*	MD	*p*	MD	*p*	MD	*p*	MD	*p*
BMI (kg/m^2^)	32.51	3.20	31.85	3.33	32.59	3.50	30.03	3.49	164.57	.001*	− 0.08	.818	1.82	.001*	0.66	.001*	2.56	.001*
FA: YFAS total	1.81	1.29	1.45	1.13	1.64	1.10	1.32	1.08	0.15	.704	0.17	.180	0.13	.301	0.36	.001*	0.32	.001*
UPPS-P (lack of) Premeditation	19.86	5.32	20.34	5.94	19.57	5.29	19.28	4.93	1.75	.187	0.29	.612	1.06	.079	− 0.48	.239	0.29	.480
UPPS-P (lack of) Perseverance	19.25	4.79	19.45	5.29	18.22	4.77	17.60	4.30	2.94	.088	1.03	.047*	1.85	.001*	− 0.20	.536	0.62	.075
UPPS-P sens. seeking	20.08	6.65	20.88	7.20	20.23	6.75	20.22	7.08	1.38	.241	− 0.15	.833	0.66	.396	− 0.80	.093	0.01	.984
UPPS-P positive urgency	23.09	7.92	24.21	9.22	22.72	7.92	21.89	7.83	4.79	.029*	0.37	.665	2.32	.012*	− 1.12	.069	0.83	.196
UPPS-P negative urgency	24.69	6.57	24.83	6.75	23.82	6.83	22.37	6.60	5.38	.021*	0.87	.233	2.46	.001*	− 0.14	.757	1.45	.004*
STROOP words	86.61	21.35	86.91	16.68	89.32	18.22	87.15	16.67	2.57	.110	− 2.71	.185	− 0.24	.882	− 0.30	.782	2.17	.051
STROOP colours	56.06	14.37	56.83	14.06	57.64	12.91	59.19	10.70	0.55	.458	− 1.58	.267	− 2.36	.066	− 0.77	.291	− 1.55	.042*
STROOP words/colours	31.63	11.48	32.53	10.88	31.81	9.62	33.04	9.10	0.16	.688	− 0.18	.861	− 0.51	.606	− 0.90	.099	− 1.23	.034*
STROOP interference	− 1.96	8.33	− 1.51	7.34	− 2.84	7.26	− 1.99	6.55	0.20	.654	0.88	.300	0.48	.509	− 0.45	.445	− 0.85	.177
CPT commissions	22.55	16.57	20.05	14.63	21.07	14.27	20.35	15.34	2.15	.144	1.48	.363	− 0.30	.846	2.50	.003*	0.72	.411
CPT hit reaction time	467.2	106.5	463.7	64.5	461.1	83.7	468.1	118.5	467.2	.387	6.04	.561	− 4.43	.665	3.48	.675	− 6.98	.423
IGT Total	2.61	21.66	5.45	22.30	2.63	22.51	1.47	23.49	1.64	.202	− 0.02	.994	3.98	.110	− 2.84	.188	1.16	.605

*SD* standard deviation, *MD* mean difference, *BMI* body mass index, *FA* food addiction.

*Bold: significant comparison (.05 level).

Regarding trait impulsivity, significant interaction parameters were found for the UPPS-P positive and negative urgency traits, indicating that changes in pre-post measures were different depending on the group of treatment (control-intervention). More specifically, patients of the intervention group presented significantly lower positive and negative urgency at post treatment than patients in the control group and, patients in the intervention group presented a significant decrease on negative urgency (pre-post) whereas the patients in the control group did not. Finally, patients in the control group presented higher scores in lack of perseverance both in the pre and post treatment.

Additionally, a tendency to decrease inhibitory control was observed through the STROOP and CPT in both groups but the intervention group reached a significant improved performance on the pre-post STROOP words/colours whereas the control group on the CPT commissions. No significant changes were found concerning trait impulsivity or choice impulsivity (decision making).

Table 4 comprises the final multiple regression results depicting the incremental capacity of FA and impulsivity measures as well as treatment group on BMI pre-post change. These results show that after adjusting by the participants’ sex, age, education and BMI at baseline, a higher decrease in the BMI was achieved for individuals with a lower score in the UPPS-P negative urgency and for those included into the intervention group.

**Table 4:** Predictive capacity of food addiction (YFAS total) and impulsivity (UPPS-P, STROOP-interference and STROOP-w/c, CPT-commissions-omissions-hit reaction times) at baseline on the BMI pre-post changes: multiple linear regression (final model).

		Coefficients						Change stat	
Model 1	Criterion: BMI pre-post	B	SE	Beta	*p*	95%CI B		∆R^2^	*p*
1^st^ block (ENTER)	Sex (0 = men; 1 = women)	− 0.087	0.154	− .026	.570	− 0.390	0.215	.042	.006*
	Age (years-old)	0.032	0.016	.088	.053	0.000	0.064		
	Education (years)	− 0.017	0.016	− .050	.280	− 0.049	0.014		
	BMI (baseline, kg/m^2^)	0.066	0.022	.132	.003*	0.022	0.110		
2^nd^ block (ENTER)	FA: YFAS total (baseline)	− 0.003	0.064	− .002	.969	− 0.129	0.124	.005	.172
3^rd^ block (STEPWISE)	UPPS-P negative urgency	− 0.028	0.011	− .113	.013*	− 0.050	− 0.006	.018	.011*
4^rd^ block (ENTER)	Group (0 = control; 1 = exper.)	1.872	0.146	.562	< .001*	1.584	2.159	.308	< .001*

*FA* food addiction.

*Bold: significant parameter (.05 level). ∆R^2^: R^2^ change. (*N* = 342).

**Figure 2:**
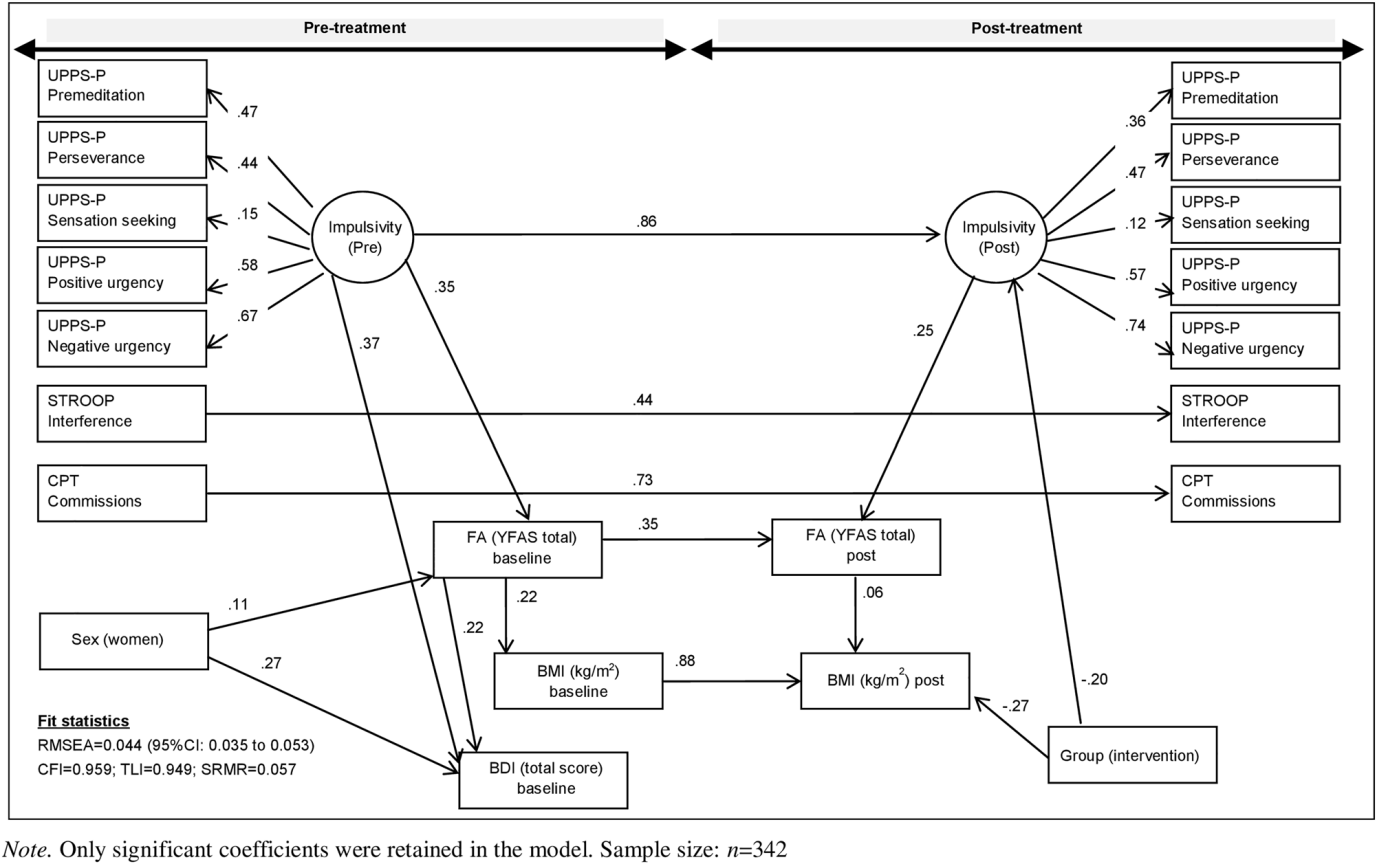
Path-diagram with the standardized coefficients in the SEM during the follow-up (adjusted by the education level).

Figure 2 displays the SEM path-diagram with the standardized coefficients (adjusted by the education level) of the variables measured during the follow-up (goodness-of-fit was achieved). The results of this model are similar to those obtained in the SEM for the cross-sectional analysis. Regarding the post-treatment results, FA scores were higher for participants who reported higher scores in the latent variable defined as trait impulsivity (UPPS-P).

Additionally, participants in the intervention group reported lower levels of trait impulsivity and lower BMI. Post treatment FA scores mediated the association between trait impulsivity and the BMI, that is, higher trait impulsivity is related to higher FA, and higher FA increases the BMI. The treatment group also achieved indirect effects on the FA and the BMI through the impulsivity trait: being in the intervention group decreases the impulsivity levels and lower impulsivity levels related to both lower FA and lower BMI.

## Discussion

This study examined if overweight and obesity in mid/old age individuals with MetS were related to specific impulsivity domains, food addiction and depressive symptoms, and if these factors can be modified by 1 year of a multimodal lifestyle intervention. Results showed the mediating role of FA between trait impulsivity and BMI or depressive symptoms at baseline and revealed that, after 1 year of treatment, BMI and some impulsivity features can be further decreased in the patients of the intervention group.

At a cross-sectional level, our results showed higher negative urgency and lack of perseverance values in individuals with higher FA and higher depressive symptoms, but no direct associations were found between impulsivity and higher BMI. Still, SEM analysis revealed that FA not only predicted higher BMI and depressive symptoms, but also achieved a mediational role between impulsivity levels and the BMI and depressive symptoms. Interestingly, negative urgency (the propensity to act rashly when facing negative emotion) has long been described as one of the main impulsivity traits linked to the development and maintenance of FA^3,26,80^ and other addictions, such as substance abuse, gambling disorder and compulsive buying^81–84^. Likewise, lack of perseverance (the inability to remain focused on a task) has been linked to higher disorder severity and poorer treatment response of addictive behaviours such as gambling or Internet addiction^85,86^. In the current sample of the study, these two impulsivity traits seem to be linked to obesity/overweight through the presence of FA. Recent studies have pointed out that FA may partly explain obesity^87^. However, it would not be the only responsible factor of obesity, which would be the result of different metabolic, behavioural or psychological factors^42,88,89^. Therefore, in our sample, only the individuals presenting higher scores in FA would also present impulsive traits long associated with addictive patterns. Finally, depressive symptoms were found to be greater for individuals with higher FA, impulsivity and BMI which suggest that the mood state of the individuals could be affected by the struggles deriving from these variables. This has previously been reported in different samples where obesity, high impulsivity and FA can hamper the individuals’ daily life and be related to a perceived worse quality of life and lower mood state^6,90^.

At a longitudinal level, after 1 year of treatment (either in the intervention group or the control group), patients reported a significant decrease in BMI and FA, together with a significant tendency to decrease different impulsivity features (i.e. trait impulsivity and inhibitory control). However, the decrease of BMI and the improvement of some impulsivity features (negative urgency and inhibitory control) were higher for the patients in the intervention group. As previously stated, it has to be noted that negative urgency is an impulsivity trait highly linked to addictive behaviours, such as FA^27^. Thus, the changes found within these variables reinforce the usefulness of an intensive multimodal psychosocial intervention (promoting a healthy dietary pattern such as the Mediterranean diet, physical activity and behavioural-motivational support) not only on reducing obesity as a risk factor for MetS and cardiovascular diseases^48,91^, but also on decreasing impulsivity and FA (source of distress and highly linked to obesity^3,43^). As previously stated, its success is higher than the one observed in the control group and seems to be largely associated with aspects of the individual's impulsivity that we can modulate through group support sessions. With this in mind, it strikes as interesting for future studies to explore the role of treatment compliance within the studied variables. For instance, to explore if a lower impulsivity is related to a better compliance and thus to better treatment outcomes.

Results also stressed that, despite the treatment arm, the higher decrease in the BMI was achieved for individuals with lower negative urgency and better inhibitory control. We hypothesise that those individuals presenting stronger psychological features related not only to FA but also to addictions in general (such as negative urgency) present a greater resistance to reduce BMI and could better benefit from a more psychologically driven intervention. In fact, previous studies have highlighted that this trait may hamper attempts to treat patients with different addictive behaviours^92,93^. Consequently, in line with this hypothesis, the SEM analysis showed that after 1 year of treatment and despite the improvement of the patients (measured by a lower BMI and FA scores), the associations between trait impulsivity and FA/BMI were still present.

The current study should be considered under some limitations. Firstly, given that our study participants are senior adults with MetS, the present findings cannot be extrapolated to other population groups. However, it is one of the strengths of this study that it is conducted with a large sample of mid/old age men and women with overweight or obesity and at high cardiovascular risk. Future studies should further explore the associations reported regarding the presence of FA, impulsivity and BMI in different populations of advanced age. Also, some of the measures are self-reported thus subject to recall biases and to complement issues. For instance, 147 participants had to be excluded due to missing data (e.g.: items not answered within one questionnaire) and the sample was reduced.

To conclude, overweight and obesity in mid/old age individuals with MetS are related to higher impulsivity, food addiction and depressive symptoms. Our results provide evidence towards the role of FA on BMI and depressive symptoms as well as its link with trait impulsivity. Finally, an intensive multimodal intervention is better than the treatment following the usual recommendations and the success is largely associated with aspects of the individual's impulsivity that we can modulate through group support sessions within the intervention. The results also highlight the modifiable nature of FA and BMI partially through improving impulsivity features and, second the need of promoting interventions within this population.

## Supplementary Information

Supplementary information.
