# Supplementary material for: Effects of a psychosocial intervention at one-year follow-up in a PREDIMED-plus sample with obesity and metabolic syndrome
Source: Sci Rep. 2021 Apr 28;11:9144. doi: 10.1038/s41598-021-88298-1 (PMC8080657; doi:10.1038/s41598-021-88298-1)

**Effects of a Psychosocial Intervention at One-year Follow-up in a PREDIMED-Plus sample with Obesity and Metabolic Syndrome**

Núria Mallorquí-Bagué^#1,2,3^, María Lozano-Madrid^#2,3^, Cristina Vintró-Alcaraz^2,3^, Laura Forcano^3,4^, Andrés Díaz-López^3,5^, Ana Galera^3,6^, Rebeca Fernández-Carrión^3,7^, Roser Granero^3,8^, Susana Jiménez-Murcia^2,3,9^, Dolores Corella^3,7,^, Xavier Pintó^3,6^, Aida Cuenca^3,4^, Mònica Bulló^3,5,10^, Jordi Salas-Salvadó^3,510,11^, Rafael de la Torre^3,4,12^*, Fernando Fernández-Aranda*^2,3,9^

**Authors affiliations:**

^1^Addictive Behaviours Unit, Department of Psychiatry, Hospital de la Santa Creu i Sant Pau, Biomedical Research Institute Sant Pau (IIB Sant Pau), Barcelona, Spain.

^2^Department of Psychiatry, University Hospital of Bellvitge-IDIBELL, Hospitalet de Llobregat, Barcelona, Spain.

^3^Consorcio CIBER, M.P. Fisiopatología de la Obesidad y Nutrición (CIBERObn), Instituto de Salud Carlos III (ISCIII), Madrid, Spain.

^4^ Integrative Pharmacology and Neurosciences Systems, Institut Hospital del Mar d’Investigacions Mèdiques (IMIM), Barcelona, Spain.

^5^ Unitat de Nutrició, Departament de Bioquímica i Biotecnologia Humana, Universitat Rovira i Virgili, Reus, Tarragona, Spain.

^6^Lipids and Vascular Risk Unit, Internal Medicine, University Hospital of Bellvitge, Hospitalet de Llobregat, Barcelona, Spain.

^7^Department of Preventive Medicine, University of Valencia, Valencia, Spain.

^8^Departament de Psicobiologia i Metodologia, Universitat Autònoma de Barcelona, Cerdanyola del Vallès, Barcelona, Spain.

^9^Department of Clinical Sciences, School of Medicine and Health Sciences, University of Barcelona, Hospitalet de Llobregat, Barcelona, Spain.

^10^Institut d'Investigació Pere Virgili (IISPV), Reus, Tarragona, Spain.

^11^University Hospital of Sant Joan de Reus, Nutrition Unit, Reus, Tarragona, Spain.

^12^Departament de Ciències Experimentals i de la Salut Universitat Pompeu Fabra (CEXS-UPF), Barcelona, Spain.

**Shared authorship:**

**^#^**Núria Mallorquí-Bagué, María Lozano-Madrid

***Corresponding authors:**

Fernando Fernandez Aranda

Department of Psychiatry

University Hospital of Bellvitge-IDIBELL

Feixa Llarga s/n

L’Hospitalet del Llobregat, Barcelona, 08907, Spain

Phone+34-93-2607227

Fax. +34-93-2607193

e-mail: ffernandez@bellvitgehospital.cat

Rafael de la Torre

Integrative Pharmacology and Neurosciences Systems

Institut Hospital del Mar d’Investigacions Mèdiques

Dr. Aiguder 88, Barcelona, 08003, Spain

Phone +34-93-3160484

Fax. +34. 93-3160467

e-mail: RTorre@imim.es


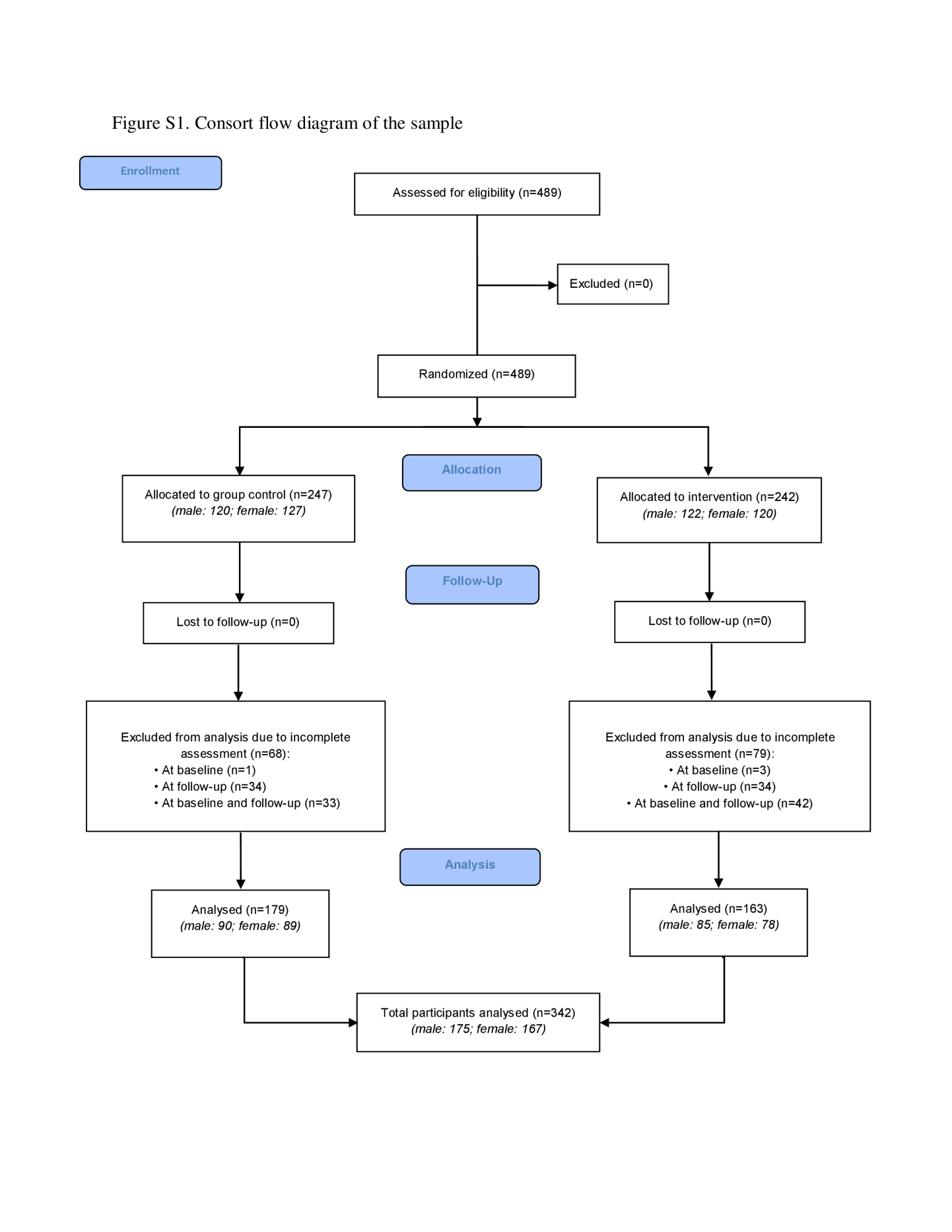

Supplement: Supplementary file 1 — Supplementary information. [file 41598_2021_88298_MOESM1_ESM.docx]
